# Occupational Health Response to SARS

**DOI:** 10.3201/eid1101.040637

**Published:** 2005-01

**Authors:** David Koh, Meng-Kin Lim, Choon-Nam Ong, Sin-Eng Chia

**Affiliations:** * National University of Singapore, Singapore

**Keywords:** letter, severe acute respiratory syndrome, occupational disease, occupational health

**To the Editor**: Severe acute respiratory syndrome (SARS), an occupational disease risk for healthcare workers, warrants an occupational health response, as clearly described by Esswein et al. (*1*). Occupational health professionals played a role in the assessment of healthcare facilities in Taiwan and many other countries. For example, occupational health professionals were invited to perform audits in at least 2 hospitals in Singapore during the height of the crisis (*2*), and to conduct follow-up discussions with the hospital management. In addition to assessment of the industrial hygiene aspects, which included evaluating the ventilation modifications needed for effective infection control, temperature and humidity were significant factors affecting the use of protective gear in a tropical country like Singapore. The occupational health audits included site inspections and reviews of work processes of those areas where actual transmission of SARS had occurred and where triage of febrile patients was taking place. Other issues identified as requiring urgent attention were providing sufficient rest, shower, and changing facilities for staff, monitoring staff sickness absenteeism, and proactively managing staff mental health. Occupational health physicians subsequently served on hospital SARS debriefing committees that reviewed institutional shortcomings and recommended new measures for future outbreaks. An occupational health service unit headed by a trained occupational health physician was formed in 1 hospital.

Other occupational groups, as well as healthcare workers, are also at potential risk. These groups may include the following: 1) food handlers, defined as persons who handle, kill, or sell food animals, and persons who prepare and serve food. (More than one third of the cases in China with onset of SARS before February 1, 2003, were in food handlers [*3*].); 2) public transportation workers and airline crew (*4*); and 3) laboratory workers handling samples or items contaminated with SARS-associated coronavirus (*5*). In Singapore, 2 taxi drivers were infected after ferrying SARS patients to healthcare facilities, and 1 Singapore Airlines cabin attendant came down with the infection after a flight with infected passengers on board. Occupationally acquired SARS infections have been documented in Singapore, Taiwan, and Beijing. Clearly, occupational health responses are needed in these occupational settings.

The recognition of SARS as an occupational disease has broader implications. Depending on country legislation, persons who contract SARS while performing their work may be eligible for worker’s compensation. Employers would be obligated to provide a safe and healthy workplace for their employees.
